# Activity patterns and interactions of rodents in an assemblage composed by native species and the introduced black rat: implications for pathogen transmission

**DOI:** 10.1186/s40850-022-00152-7

**Published:** 2022-08-26

**Authors:** Rodrigo Salgado, Isabel Barja, María del Carmen Hernández, Basilio Lucero, Ivan Castro-Arellano, Cristian Bonacic, André V. Rubio

**Affiliations:** 1 Departamento de Ciencias Biológicas Animales, Facultad de Ciencias Veterinarias Y Pecuarias, Universidad de Chile, Santiago, Chile; 2 Departamento de Biología, Unidad de Zoología, Campus Universitario de Cantoblanco, Universidad Autónoma de Madrid, Madrid, España; 3 Centro de Investigación en Biodiversidad Y Cambio Global (CIBC-UAM), Campus Universitario de Cantoblanco, Universidad Autónoma de Madrid, Madrid, España; 4 Biology Department, Texas State University, San Marcos, USA; 5 Departamento de Ecosistemas Y Medio Ambiente, Facultad de Agronomía E Ingeniería Forestal, Pontificia Universidad Católica de Chile, Santiago, Chile

**Keywords:** Chile, Interspecific interactions, Rodentia, Temporal overlap, Wild reservoirs

## Abstract

**Background:**

The degree of temporal overlap between sympatric wild hosts species and their behavioral interactions can be highly relevant to the transmission of pathogens. However, this topic has been scantly addressed. Furthermore, temporal overlap and interactions within an assemblage of wild rodents composed of native and introduced species have been rarely discussed worldwide. We assessed the nocturnal activity patterns and interactions between rodent taxa of an assemblage consisting of native species (*Oligoryzomys longicaudatus*, *Abrothrix hirta*, and *Abrothrix olivaceus*) and the introduced black rat (*Rattus rattus*) in a temperate forest from southern Chile. All rodent species in this study are known hosts for various zoonotic pathogens.

**Results:**

We found a high nocturnal temporal overlap within the rodent assemblage. However, pairwise comparisons of temporal activity patterns indicated significant differences among all taxa. *Rattus rattus* showed aggressive behaviors against all native rodents more frequently than against their conspecifics. As for native rodents, agonistic behaviors were the most common interactions between individuals of the same taxon and between individuals of different taxa (*O. longicaudatus* vs *Abrothrix* spp.).

**Conclusions:**

Our findings reveal several interactions among rodent taxa that may have implications for pathogens such as hantaviruses, *Leptospira* spp., and vector-borne pathogens. Furthermore, their transmission may be facilitated by the temporal overlap observed between rodent taxa.

**Supplementary Information:**

The online version contains supplementary material available at 10.1186/s40850-022-00152-7.

## Introduction

Rodentia is the most diverse order of mammals and is composed mainly of species of small size and home range [1]. Within a relatively small area, it is possible to find many species of rodents that potentially interact with each other. Consequently, this group has often been used to understand the organization of communities [2–4]. Furthermore, several rodents are also recognized as reservoirs of zoonotic pathogens [5]. Since interspecific contacts and competition within host communities can be very relevant to determining the transmission of pathogens [6], it is of interest to understand the degree of temporal overlap between rodent species and their interactions. Species interactions are especially relevant for directly transmitted pathogens such as rodent-borne hantavirus, where co-occurring non-reservoir rodents may influence contact rates between infected and susceptible reservoir rodents, modulating pathogen transmission [7–9].

Within a community, species distribute resources to coexist, whether in the spatial, food, or temporal dimension [10]. The time axis has been recognized as a less common partition resource. However, there is growing evidence demonstrating the ecological significance of time use to understand relationships among species [11–17]. For some rodent communities, temporal segregation might help in allowing coexistence between species [18–24]. On the other hand, other rodent communities have shown a high temporal overlap [8, 25].

In the temperate forest of southern Chile, the most abundant native rodent species such as the long‐tailed colilargo (*Oligoryzomys longicaudatus*), the shaggy soft-haired mouse (*Abrothrix hirta*), and the olive grass mouse (*Abrothrix olivaceus*), are recognized as hosts of zoonotic pathogens such as Andes Orthohantavirus, *Leptospira* spp. and *Bartonella* spp. [26–28]. In addition, the invasive black rat (*Rattus rattus*) is commonly found in this temperate forest [29–31]. *Rattus rattus* was introduced more than 300 years ago in Chile and has colonized many natural areas [29]. This rodent is also associated with several zoonotic pathogens in Chile and globally [27, 28, 32, 33], and shares parasites with native Chilean rodents, suggesting interspecies transmission [34]. Furthermore, *R. rattus* in other world areas exerts a significant negative impact on communities of small mammals and other native species through competitive interactions and predation [35, 36]. Consequently, the study of its interaction with native rodents is relevant for understanding its impact on native fauna and pathogen transmission. However, there is scarce research, especially on the temporal overlap of activity and interactions between native rodents and *R. rattus* around the world [37].

Here we conducted a field study in a temperate forest in southern Chile to assess the activity patterns and interactions between rodent taxa of an assemblage of rodents composed of native species and *R. rattus*. We then discussed the potential implications of our findings concerning pathogen transmission among rodent hosts.

## Materials and methods

### Study area

The study was carried out in a temperate forest located in Huelemolle (39º16’S, 71º48’ W), Araucanía Region, southern Chile. The climate in this zone is temperate-humid with a short dry season (< 4 months) in summer (January–March) and an average yearly rainfall of 2000 mm [38]. Forests are dominated by Patagonian oak (*Lophozonia obliqua*) and coigue (*Nothofagus dombeyi*), mainly associated with Chilean laurel (*Laurelia sempervirens*), olivillo (*Aextoxicon punctatum*), lingue (*Persea lingue*), and ulmo (*Eucryphia cordifolia*) [39].

### Rodent species

According to live-trapping surveys in the study area conducted during autumn, winter, and summer [30, 40, 41], the assemblage of rodents is composed of four species: *A. hirta* (until 2014 considered a synonym of the long-haired field mouse, *A. longipilis* [42]), *A. olivaceus,* the *O. longicaudatus*, and the introduced *R. rattus* (Supplemental Table S1). *Abrothrix hirta* is a sigmodontine medium-sized rodent (body length ~ 107 mm; mean body mass 41.2 g) [40], of gray color with brownish hues. It is terrestrial and omnivorous. This species inhabits a wide range of habitats, including sclerophyllous forests, shrublands, temperate rainy forests, arid steppes east of the Andes, and can be found near rural settlements [43]. *Abrothrix olivaceus* is a small sigmodontine rodent (body length ~ 78 mm, mean body mass 20.4 g) [40], of a dorsal color generally grayish with brownish-olive-colored streaks. It is terrestrial and omnivorous. This species inhabits a wide range of habitats such as stony coastal deserts, thorn scrubs and rainy temperate forests. It is one of the Chilean species that best adapts to anthropized environments [43–45]. *Oligoryzomys longicaudatus* is small sigmodontine rodent (body length ~ 83.8 mm; mean body mass 22.8 g) [40], with dorsal coloration light brown to slightly darker tones. It is a scansorial rodent (i.e., ability or propensity to climb), and omnivorous. It is considered very versatile in its selection of habitat, being found from wooded areas to ecotonal areas between forests and shrublands, but with a preference for humid areas. It is also found in rural settlements [43]. *Rattus rattus* is larger than these native rodents (body length ~ 163 mm; mean body mass 113.4 g) and is considered scansorial and omnivorous [43].

### Rodent activity and interactions

To assess rodent activity and interactions, we conducted camera-trapping surveys. Camera traps are increasingly used in small mammal research [46], including studies on activity patterns [8, 15, 23, 47–50] and behaviors [8, 30, 51, 52]. This passive method allows the collection of data on both activity patterns and interaction behaviors of nocturnal forest-dwelling rodents under natural conditions, which is not possible with other methods such as live trapping and direct visualization.

Camera trapping has been conducted in the study area for different ecological and behavioral studies targeting mainly *O. longicaudatus*. We retrieved data recorded during May 2019 (austral autumn) and January 2021 (austral summer) for this specific work. Due to restrictions caused by the COVID-19 pandemic, data could not be obtained in 2020. In autumn, we placed 48 sampling stations separated by at least 25 m, while in summer, we installed 24 stations a minimum of 20 m apart. We used these distances between cameras because according to our live trapping survey in the area, the native species (*A. hirta, A. olivaceus,* and *O. longicaudatus*) move a smaller distance in short periods of days (4 days). In addition, given the abundance recorded in our live captures (Supplemental Table S1), we expected to record videos of several individuals on each camera. In both seasons, the sampling stations were in the same study area but not strictly at the same point. Each sampling station consisted of one passive infrared camera trap (Bushnell Trophy Cam, 119537C, Bushnell Optics, Overland Park, Kansas) mounted horizontally 1.5 m above the ground on a PVC pipe [53] and baited with oats and vanilla essence placed in a plastic plate at ground level. We used this distance and orientation of the cameras, similar to Rendall et al. [53], because it allows us to have a sufficient area of the ground (0.89 m^2^) to analyze rodent interactions in foraging arenas, and also a dorsal view of the rodents, which provides us with a better view of body and tail proportions that help us identify rodent species.

The cameras were configured to record 30 s videos at each activation, with 0.6 s intervals. It continued recording as long as at least one individual stayed in front of the sensor. The medium sensitivity setting was applied to all cameras, and two layers of masking tape were added to the cameras to reduce flash brightness for close-range operation. Cameras were activated from 5:00 pm to 9:00 am for 3 or 4 consecutive nights at each sampling station since rodents in the study area are crepuscular-nocturnal [54]. In addition, our pilot tests of camera traps in the study sites did not record rodent activity during the day (Supplemental Material). We registered the date, time, and species for each video recorded. Video recordings did not allow reliable identification of specific individuals. Therefore, all analyses were at the taxa level (see more details below). Previous training in live rodent trapping in the study area allowed us to reliably identify *R. rattus* and *O. longicaudatus* in the videos. For rodents of the genus *Abrothrix* (*A. hirta* and *A. olivaceus*), it was difficult to identify both species in all recordings. Therefore, we combined their records as *Abrothrix* spp. Due to this limitation, three rodent taxa were considered for the analysis of activity patterns and interactions: (1) *Abrothrix* spp., (2) *O. longicaudatus*, and (3) *R. rattus*. We organized video recordings by "events" to distinguish between independent detections of each taxon. An event was considered as detection(s) of the same taxon within a five-minute interval [15, 30].

When more than one individual was present in the same 30-s video, their interactions were classified into four categories of behavior, similarly to Scheibler et al. [55]: a) *Sociopositive:* when the animals were contact-sitting (animals sitting side by side in close body contact with each other), allogrooming (individuals grooming each other), or showing courtship behavior (e.g., mounting, anogenital contacts, circling); b) *Neutral*: when animals were only feeding, sitting and resting or autogrooming (individual grooming itself); c) *Aggressive*: including biting (an animal attacked by another), and chasing (one animal trying to escape with the aggressor following closely), and d) *Agonistic*: keeping distance (animals hiding or running away when the oncoming animal appeared).

### Data analysis

We analyzed temporal activity patterns using circular statistics. For each rodent taxon, we first calculated the mean vector (*µ*), i.e. direction of mean angle, and the mean vector length (*r*), i.e. a measure of concentration that varies from 0 (when there is no concentration of data) to 1 (when all data are concentrated at the same direction) [56]. Circular histograms were drawn to visualize the frequency of events at each 1-h period. The uniformity of the nocturnal activity of each taxon was evaluated through the Rayleigh uniformity test [56], which assesses whether the independent events of each taxon were randomly or uniformly distributed. To compare temporal activity patterns between taxa and between seasons, we used pairwise comparisons calculating the W test statistic of the Mardia-Watson-Wheeler test [56, 57]. These statistical analyses were conducted in Oriana 4.02 software [58].

We also analyzed assemblage-wide time overlap for rodents following the analytical method of Castro‐Arellano et al. [59]. This analysis consists of a null model approach that uses Monte Carlo simulations to generate null distributions of overlap among all taxa. Ten thousand simulations were performed using a randomization algorithm (Rosario) designed specifically for temporal data. Null models have been widely used in ecology to test overlap in resource use, but randomization algorithms used for non-ordered resources (e.g., food categories or prey items) cannot be used to assess time overlap as these destroy temporal autocorrelation, a unique characteristic of use of time by most species. Instead, the Rosario algorithm not only maintains temporal autocorrelation of empirical data but also creates biologically realistic time use possibilities, thus creating an adequate null space for contrast to empirical data. Significance is determined by comparison of randomized overlap values to the amount of empirical overlap. The basis of each analysis was a matrix of the relative number of events for each rodent taxon during each time 1-h interval. Observed overlap was quantified as the average of all pairwise overlap values calculated via the Czechanowski index [60]. This empirical index was compared against a null distribution of assemblage-wide temporal overlaps generated by the Time Overlap program (freely available at: http://hydrodictyon.eeb.uconn.edu/people/willig/Research/activity%20pattern.html). This webpage not only provides access to the program but also provides explanations of its implementation. The exact details of the algorithm, as well as a series of bench tests, are described in Castro-Arellano et al. [59].

### Ethics statement

Rodent live trapping conducted in previous training for rodent identification was authorized by Servicio Agrícola y Ganadero (SAG; Chilean Fish and Wildlife Service) under permit No. 7479/2018. Bioethical approval (No. 18197-VETUCH) was issued by the Faculty of Animal and Veterinary Sciences, University of Chile.

## Results

### Temporal activity

We obtained 4,474 events to analyze the temporal activity patterns and temporal overlap. The following taxa were represented (percentage of events in parentheses): *Abrothrix* spp. (51.7%), *R. rattus* (29.4%), and *O. longicaudatus* (18.9%). Events and descriptive statistics by each taxon are detailed in Table 1.

**Table 1:** Descriptive circular statistics of temporal activity pattern for rodents in a temperate forest from Araucanía Region, Chile

	*Oligoryzomys longicaudatus*	*Abrothrix* spp.		*Rattus rattus*	
	Autumn *n* = 845	Autumn *n* = 1686	Summer *n* = 626	Autumn *n* = 1050	Summer *n* = 267
Mean Vector (*µ*)	23:49 (357.30°)	23:11 (347.79°)	01:51 (27.98°)	00:11 (2.87°)	02:16 (34.20°)
Length of Mean Vector (*r*)	0.62	0.64	0.83	0.60	0.83
Circular Variance	0.38	0.36	0.17	0.40	0.17
Mode hour	23:00–00:00 (9.80%)	19:00–20:00 (11.92%)	04:00–05:00 (14.00%)	19:00–20:00 (11.62%)	04:00–5:00 (20.97%)
Onset activity hour	18:00–19:00 (7.10%)	17:00–18:00 (0.12%)	21:00–22:00 (1.43%)	18:00–19:00 (4.40%)	21:00–22:00 (0.36%)
Offset activity hour	07:00–08:00 (0.47%)	07:00–08:00 (1.36%)	06:00–07:00 (0.32%)	07:00–08:00 (0.09%)	06:00–07:00 (0.75%)
Rayleigh Test (*Z*)	327.90	694.60	433.90	381.10	185.70
Rayleigh Test (*P*)	< 0.001	< 0.001	< 0.001	< 0.001	< 0.001

**Fig. 1:**
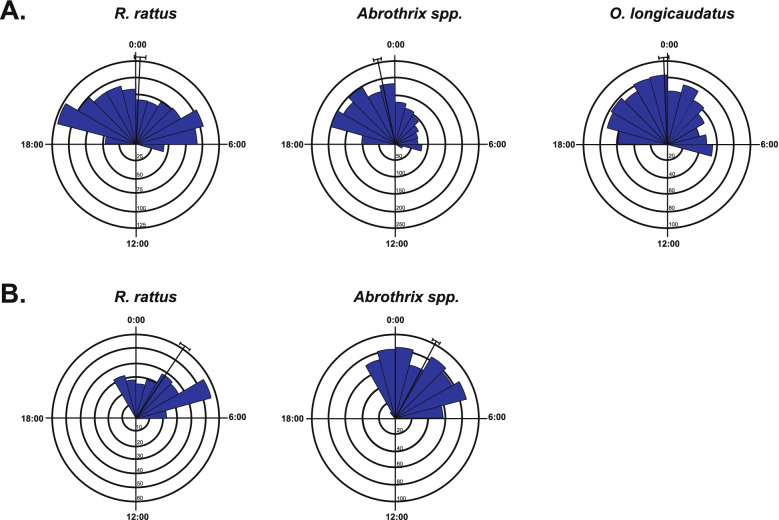
Activity patterns of three taxa in a temperate forest from Araucanía Region, Chile: **A**) Autumn season; **B**) Summer season. Bars represent the number of independent records (n) per hour, and the internal circles indicate the frequency value. The radius indicates the mean vector with a curved line representing the 95% confidence interval for the mean

According to the Rayleigh test, no uniformity in nocturnal activity was found for any rodent taxon (Table 1). As indicated by the Mardia-Watson-Wheeler test, pairwise comparisons of temporal activity patterns showed significant differences among all taxa (Table 2, Fig. 1). Nocturnal activity was also different between seasons for the *R. rattus* (*W* = 333.9, *P* < 0.001) and for *Abrothrix* spp. (*W* = 121.7, *P* < 0.001), with a reduction in activity time in summer compared to autumn, starting later, and ending earlier (Table 1; Fig. 1). No seasonal comparison was conducted for *O. longicaudatus*, as it was not recorded in summer. Assemblage-wide activity overlap was highly consistent among taxa in both seasons. There was a larger assemblage-wide temporal overlap in all cases than the random expectation (Table 3).

**Table 2:** Pairwise comparisons of temporal activity patterns between taxa, using Mardia-Watson-Wheller tests (*W*). Analyses were conducted separately by season (Autumn and Summer)

Season	Taxa (events)	*W*	*P*
Autumn	A.B (1686) vs R.R (1050)	37.72	< 0.001
	A.B (1686) vs O.L (845)	20.61	< 0.001
	R.R (1050) vs O.L (845)	6.32	0.042
Summer	A.B (626) vs R.R (267)	7.23	0.027

*A.B* *Abrothrix* spp., *O.L*  *O. longicaudatus*, *R.R* *R. rattus*

**Table 3:** Results of ROSARIO algorithm null model analyses of temporal niche overlap (Czekanowski index) in rodent assemblage of a temperate forest from Araucanía Region, Chile, for autumn and summer seasons

Season	Taxa number	Observed overlap	ROSARIO	
			Simulation overlap (*SD*)	*P* value
Autumn	3	0.87	0.72 (0.03)	0.002
Summer	2	0.91	0.47 (0.24)	0.034

### Rodent interactions

We registered 314 events of interactions between individuals of the same taxon and 253 events of interactions between individuals of different taxa to analyze behaviors.

*Rattus rattus* showed aggressive behavior against native rodents more frequently, followed by agonistic interactions (Table 4). In contrast, neutral and agonistic behaviors were the most common intraspecific interactions of *R. rattus* (Table 5). Regarding the native rodents, agonistic behavior was observed more frequently between *O. longicaudatus* and *Abrothrix* spp. (Table 6). Agonistic behavior was also the most common interaction between *O. longicaudatus* individuals and between *Abrothrix* spp. individuals. Aggressive and neutral interactions were also frequently observed (Table 6). Because the number of interactions was very unbalanced between seasons for most taxa, analyses comparing behaviors were not performed.

**Table 4:** Frequency (%) of observed behaviours between *R. rattus* and native rodents (*O. longicaudatus* and *Abrothrix* spp.) during Autumn 2019 and Summer 2021

Categories of Behaviors	Specific interactions	*Oligoryzomys longicaudatus*	*Abrothrix* spp.	
		Autumn 2019 *n* = 12	Autumn 2019 *n* = 26	Summer 2021 *n* = 21
Aggressive	Attacks …	50%	58%	48%
Aggressive	Is attacked by …	0%	4%	5%
Agonistic	Disappears when … arrives	25%	8%	0%
Agonistic	Causes disappearance of …	17%	27%	24%
Neutral	Neutral behavior with …	8%	4%	24%
Socio-positive	Positive behavior with …	0%	0%	0%

*n* Number of interaction events

**Table 5:** Frequency (%) of interactions of individuals of the same taxon of rodents according to each behaviour

	*Oligoryzomys longicaudatus*	*Rattus rattus*		*Abrothrix* spp.	
Behaviors	Autumn *n* = 53	Autumn *n* = 42	Summer *n* = 11	Autumn *n* = 124	Summer *n* = 14
Aggressive	11%	24%	27%	29%	50%
Agonist	77%	7%	45%	52%	7%
Neutral	11%	50%	9%	18%	29%
Positive	0%	19%	18%	2%	14%

*n* Number of interaction events

**Table 6:** Frequency of observed behaviors between *O. longicaudatus* (O.L) and *Abrothrix* spp. (A.B) during Autumn 2019 (*n* = 194 interaction events)

Categories of Behaviors	Specific interactions	Frequency (%)
Aggressive	O.L attacks A.B	4%
Aggressive	O.L is attacked by A.B	5%
Agonistic	O.L disappears when A.B arrives	47%
Agonistic	O.L causes the disappearance of A.B	38%
Neutral	Neutral behaviors between O.L and A.B	6%
Sociopositive	Positive behaviors between O.L and A.B	0%

## Discussion

Our study recorded the nocturnal foraging activity of a rodent community composed of native species and an introduced one (*R. rattus*), in a temperate forest of southern Chile. We observed a high temporal activity overlap of the rodent assemblage, but some differences in the use of time for each rodent taxon and seasons were observed.

In autumn, *O. longicaudatus* and *Abrothrix* spp. showed more significant activity during the first half of the night. But *O. longicaudatus* showed a more distributed use across hours. Something similar was observed by Delibes-Mateos et al. [61] in a community of small mammals with the same native species and other rodent species within a Valdivian temperate forest in southern Chile. They described a unimodal pattern, with a maximum peak before midnight. However, in their study, researchers did not separate activity patterns by rodent species or taxa. Regarding *R. rattus*, the activity in autumn was bimodal, close to twilight and before dawn, consistent with what was reported by Whisson et al. [62] for this species in an old-growth riparian forest in California. They observed a heightened activity 1 h before sunrise and 1–2 h after sunset. In contrast, during the summer, as nighttime is reduced, our findings showed a more distributed time-use with respect to autumn for *Abrothrix* spp., and a unimodal distribution before dawn was reported for *R. rattus.* The absence of *O. longicaudatus* in summer was also expected because this species presents a significant fluctuation during the year, with complete or almost total disappearance in spring–summer [63–66].

The high level of temporal overlap within the rodent assemblages suggests that temporal partitioning would not be a resource to facilitate their coexistence. Competition between *O. longicaudatus* and *Abrothrix* spp. does not seem to be strong [67], whereas other mechanisms such as a different use of microhabitats or food habits could be enough. In forests, *O. longicaudatus* and *A. olivaceus* have shown differences at the microhabitat level associated with their mode of locomotion and as an anti-predatory mechanism [68]. On the one hand, *O. longicaudatus* is associated with foliage and shrub density variables, using sites with more shrubs and tree canopy vegetation. On the other hand, *A. olivaceus* tends to prefer forest sites with a higher volume of ground cover [68]. In contrast, *O. longicaudatus* and *A. hirta* prefer similar habitats, with differences at finer scales [66, 69]. These native rodents show remarkable plasticity regarding their feeding habits, having mixed diets among different locations. Both *Abrothrix* species have an omnivore habit [70, 71]. *Oligoryzomys longicaudatus* is often described as a seed-eating species [72–74], but it can also feed on plants and incorporate arthropods. The diet of all native rodents also varies between seasons [75–77]. Therefore, the diverse diet of these rodents can contribute to their coexistence.

The introduced *R. rattus* is a rodent with a greater capacity to adapt to different environments. Although it is recognized as a generalist in the use of habitats, in forests it has been observed a significant attraction to areas with dense understory and thick leaf litter [78, 79]. It feeds mainly on fruit and seed, with plant material often comprising 75–80% of its diet [80–82]. However, it is an opportunistic animal that can incorporate eggs and other small animals into its diet, including rodents [24, 83, 84], negatively impacting native rodents. Despite the global distribution of *R. rattus*, few studies on the effects of this species on rodent assemblages have been conducted. Most studies have focused on islands, showing *R. rattus* as a dominant competitor in rodent assemblages in New Zealand and Hawaii [85, 86]. In addition, Stokes et al. [87] in Australia and Harris and Macdonald [88] in Galapagos have shown that *R. rattus* competes with the Australian bush rat (*Rattus fuscipes*) and the Santiago Galapagos mouse (*Nesoryzomys swarthi*) respectively, mainly through interference rather than resource competition. Our findings revealed that the interactions of *R. rattus* with native rodents are primarily aggressive. Therefore, the *R. rattus* is an aggressively dominant species within the rodent assemblage. In addition, Guzmán et al. [84] recorded the remains of hairs and teeth of *O. longicaudatus* in the stomach contents of *R. rattus* from Central Chile, suggesting predation upon this native rodent. Besides these studies, no further research has been conducted on *R. rattus* and its interactions or effects on rodent assemblages in these temperate forests. Therefore, more studies are necessary to understand this species' impact in the rodent assemblage of the southern cone of America.

The interaction between rodent species within the assemblage can have consequences in the transmission of pathogens. Our findings reveal several interactions among rodent taxa that may have implications for pathogen transmission. The Andes virus (ANDV) is an important rodent-borne zoonosis, causing hantavirus cardiopulmonary syndrome in humans in Chile and southern Argentina [26]. The main reservoir is *O. longicaudatus,* and transmission among rodent individuals is supposed to be mainly by direct contacts, through aggressive encounters [89], similar to other hantaviruses in the Americas [90]. Although we found that the frequency of interspecific interactions is low compared to the total number of rodent events recorded at foraging stations, we highlight that *O. longicaudatus* interacts in ways that might imply pathogen transmission during foraging. For example, several studies have reported individuals of *A. hirta* and *A. olivaceus* seropositive to ANDV in Chile and Argentina (e.g., [26, 91, 92]). The seropositivity of both species might be a consequence of spillover events from infected *O. longicaudatus* individuals [91, 93]. In another study, Rubio et al. [94] found a higher ANDV seroprevalence in *Abrothrix* spp. from central Chile within areas with higher ANDV seroprevalence in *O. longicaudatus*, which supports the hypothesis of spillover events. Therefore, the behavioral observations of interspecific encounters like aggressive interactions can be opportunities for cross-species transmission of pathogens. Nevertheless, our findings displayed that the main encounters among *O. longicaudatus* and *Abrothrix* spp. are agonists, which do not imply direct contact. Therefore, there is less probability of transmission of pathogens such as hantaviruses. However, a close approach between individuals (not involving direct contact) can facilitate ectoparasite transmission such as fleas. For example, Moreno-Salas et al. [44] reported several flea species shared between the *O. longicaudatus* and *Abrothrix* spp. These fleas can be vectors of pathogens such as rickettsiae and bartonellae [28, 44, 95].

*Rattus rattus* has transmitted and shared numerous parasites and pathogens to native rodents worldwide [33]. In Chile, several endoparasites and ectoparasites (introduced and native) are reported in *R. rattus*, *Abrothrix* spp*.* and *O. longicaudatus*, suggesting interspecific transmission [34, 96]. In fact, these native rodents and *R. rattus* belong to the same suborder (Myomorpha), which may facilitate the transmission of parasites and pathogens among them [34, 96]. Although the frequency of aggressive interactions between *R. rattus* and native rodents were few, the observed aggressive behavior of *R. rattus* towards native rodents, and the possible occasional predation upon them [84], can facilitate parasite and pathogens transmission. For example, aggressive and predation behaviors can increase the interaction with fluids (e.g., saliva, urine) between *R. rattus* and *O. longicaudatus* individuals infected with ANDV, facilitating spillover events. In fact, *R. rattus* can be infected with ANDV, although the role of this species in ANDV transmission is unknown [29]. Pathogens such as *Leptospira spp*., which is transmitted through urine, is another example of potential cross-species transmission, and commonly found in native rodents and *R. rattus* in Chile [27, 97]. In summary, *R. rattus* in this area interact with native rodents and may generate several spillover and spillback events, potentially becoming an invasive species with a relevant role as a reservoir, even for endemic pathogens [98].

We acknowledge the limitations of this study in that we were unable to separate both species of *Abrothrix* in the analyses. Other studies using camera traps have the same limitation when some rodent species are morphologically similar [52, 99]. Future studies should use a combination of methods such as cameras and a system of transponder (PIT) tags in rodents and PIT antenna connected to data loggers [100]. These combined techniques would allow identification of species as well as individuals. However, the latter methodology is costly, and thus could not be used in this study. Another limitation was that we only analyzed rodent interactions at foraging stations. Interactions during foraging are possible opportunities for pathogen transmission between small mammals, as they may fight for food resources [101]. However, placing food resources as bait may also artificially increase the level of interactions and competition. Therefore, future experiments analyzing rodent interactions should also include other areas in the forest without including artificial food resources. For example, rodents may compete for refugia, and thus placing camera traps near burrows may provide additional information on behavioral interactions.

## Conclusions

According to our results, rodents in the temperate forest of southern Chile show a high temporal overlap, but specific temporal activity patterns demonstrate differences among all taxa. The invasive *R. rattus* are clearly aggressive towards all native rodents, which could have negative effects on native species, deserving further investigation. In addition, this study reveals several interactions between rodent species that may have implications for the transmission of directly transmitted pathogens and for vector-borne pathogens, which should also be facilitated by the temporal overlap observed between species.

## Supplementary Information

**Additional file 1.**

**Additional file 2.**

**Additional file 3.**

## Data Availability

Data generated or analyzed during this study are included in this published article [and its supplementary information files].

## References

[1] Burgin CJ, Colella JP, Kahn PL, Upham NS (2018). How many species of mammals are there?. J Mammal.

[2] Bowers MA, Brown JH (1982). Body Size and Coexistnce in Desert Rodents: Chance or Community Structure?. Ecology.

[3] Kotler BP, Brown JS (1988). Environmental heterogeneity and the coexistence of desert rodents. Annu Rev Ecol Syst.

[4] Dayan T, Simberloff D (1994). Morphological relationships among coexisting heteromyids: an incisive dental character. Am Nat.

[5] Han BA, Kramer AM, Drake JM (2016). Global patterns of zoonotic disease in mammals. Trends Parasitol.

[6] O’Regan SM, Vinson JE, Park AW (2015). Interspecific contact and competition may affect the strength and direction of disease-diversity relationships for directly transmitted microparasites. Am Nat.

[7] Dearing MD, Clay C, Lehmer E, Dizney L (2015). The roles of community diversity and contact rates on pathogen prevalence. J Mammal.

[8] Rubio AV, Castro-Arellano I, Mills JN, List R, Avila-Flores R, Suzán G (2017). Is species richness driving intra-and interspecific interactions and temporal activity overlap of a hantavirus host? An experimental test. PLoS One.

[9] Eleftheriou A, Kuenzi AJ, Luis AD (2021). Heterospecific competitors and seasonality can affect host physiology and behavior: key factors in disease transmission. Ecosphere.

[10] Schoener TW (1974). Resource Partitioning in Ecological Communities: Research on how similar species divide resources helps reveal the natural regulation of species diversity. Science.

[11] Kronfeld-Schor N, Dayan T (2003). Partitioning of time as an ecological resource. Annu Rev Ecol Evol Syst.

[12] Castillo-Ruiz A, Paul MJ, Schwartz WJ (2012). In search of a temporal niche: social interactions. Prog Brain Res.

[13] Monterroso P, Alves PC, Ferreras P (2013). Catch me if you can: diel activity patterns of mammalian prey and predators. Ethology.

[14] Monterroso P, Alves PC, Ferreras P (2014). Plasticity in circadian activity patterns of mesocarnivores in Southwestern Europe: implications for species coexistence. Behav Ecol Sociobiol.

[15] Diete RL, Meek PD, Dickman CR, Lisle A, Leung LK-P (2017). Diel activity patterns of northern Australian small mammals: variation, fixity, and plasticity. J Mammal.

[16] Gómez-Ortiz Y, Monroy-Vilchis O, Castro-Arellano I (2019). Temporal coexistence in a carnivore assemblage from central Mexico: temporal-domain dependence. Mamm Res.

[17] Nagy-Reis MB, Iwakami VH, Estevo CA, Setz EZ (2019). Temporal and dietary segregation in a neotropical small-felid assemblage and its relation to prey activity. Mamm Biol.

[18] Ziv Y, Abramsky Z, Kotler BP, Subach A (1993). Interference competition and temporal and habitat partitioning in two gerbil species. Oikos.

[19] Vieira EM, Baumgarten LC (1995). Daily activity patterns of small mammals in a cerrado area from central Brazil. J Trop Ecol.

[20] Jones M, Mandelik Y, Dayan T (2001). Coexistence of temporally partitioned spiny mice: roles of habitat structure and foraging behavior. Ecology.

[21] Castro-Arellano I, Lacher TE (2009). Temporal niche segregation in two rodent assemblages of subtropical Mexico. J Trop Ecol.

[22] Gutman R, Dayan T (2005). Temporal partitioning: an experiment with two species of spiny mice. Ecology.

[23] Viviano A, Scarfò M, Mori E (2022). Temporal Partitioning between Forest-Dwelling Small Rodents in a Mediterranean Deciduous Woodland. Animals.

[24] Mori E, Ferretti F, Fattorini N (2019). Alien war: Ectoparasite load, diet and temporal niche partitioning in a multi-species assembly of small rodents. Biol Invasions.

[25] Vieira EM, Paise G (2011). Temporal niche overlap among insectivorous small mammals. Integr Zool.

[26] Torres-Pérez F, Palma RE, Boric-Bargetto D, Vial C, Ferrés M, Vial PA (2019). A 19 year analysis of small mammals associated with human hantavirus cases in Chile. Viruses.

[27] Luna J, Salgado M, Tejeda C, Moroni M, Monti G (2020). Assessment of risk factors in synanthropic and wild rodents infected by pathogenic Leptospira spp. Captured in southern Chile. Animals.

[28] Müller A, Gutiérrez R, Seguel M, Monti G, Otth C, Bittencourt P (2020). Molecular survey of Bartonella spp. in rodents and fleas from Chile. Acta Trop.

[29] Lobos G, Ferres M, Palma RE (2005). Presencia de los géneros invasores *Mus* y *Rattus* en áreas naturales de Chile: un riesgo ambiental y epidemiológico. Rev Chil Hist Nat.

[30] Hernández MC, Jara-Stapfer DM, Muñoz A, Bonacic C, Barja I, Rubio AV (2021). Behavioral responses of wild rodents to owl calls in an Austral temperate forest. Animals.

[31] Zúñiga A, Muñoz-Pedreros AA, Quintana V. Seasonal Variation in a Small-Mammal Assemblage in a Priority Site for Conservation in South-Central Chile. Zoodiversity. 2021;55(5) 10.15407/zoo2021.05.395.

[32] Kosoy M, Khlyap L, Cosson J-F, Morand S (2015). Aboriginal and invasive rats of genus *Rattus* as hosts of infectious agents. Vector Borne Zoonotic Dis.

[33] Morand S, Bordes F, Chen HW, Claude J, Cosson JF, Galan M (2015). Global parasite and Rattus rodent invasions: the consequences for rodent-borne diseases. Integr Zool.

[34] Landaeta-Aqueveque C, del Rosario RM, Henríquez A, Yáñez-Meza A, Correa JP, González-Acuña D (2018). Phylogenetic and ecological factors affecting the sharing of helminths between native and introduced rodents in Central Chile. Parasitology.

[35] Banks PB, Hughes NK (2012). A review of the evidence for potential impacts of black rats (*Rattus rattus*) on wildlife and humans in Australia. Wildl Res.

[36] Shiels AB, Pitt WC, Sugihara RT, Witmer GW. Biology and impacts of Pacific island invasive species. 11. *Rattus rattus*, the black rat (Rodentia: Muridae). Pac Sci. 2014;68(2):145–84 10.2984/68.2.1.

[37] Harris DB, Gregory SD, Macdonald DW (2006). Space invaders? A search for patterns underlying the coexistence of alien black rats and Galápagos rice rats. Oecologia.

[38] Di Castri F, Hajek ER (1976). Bioclimatología de Chile.

[39] La GR (1994). vegetación natural de Chile: clasificación y distribución geográfica.

[40] Forero L. Presence and abundance of *Rattus rattus* and its relationship with composition and structure of the assemblage of native small mammals in the temperate Andean rainforest in southern Chile. Thesis, Master in Natural Resources. Santiago: Pontificia Universidad Católica de Chile; 2014.

[41] Hernández MC, Rubio AV, Barja I. Long-Tailed Pygmy Rice Rats modify their behavioural response and faecal corticosterone metabolites in response to culpeo fox but not to lesser grison. Animals. 2021;11(11):3036. 10.3390/ani11113036. 10.3390/ani11113036 PMC8614544 34827769

[42] Teta P, Cañón C, Patterson BD, Pardiñas UF (2017). Phylogeny of the tribe Abrotrichini (Cricetidae, Sigmodontinae): integrating morphological and molecular evidence into a new classification. Cladistics.

[43] Muñoz-Pedreros A, Gil C, Muñoz Pedreros A, Yáñez Valenzuela J (2009). Rodentia. Mamíferos de Chile.

[44] Moreno-Salas L, Espinoza-Carniglia M, Lizama-Schmeisser N, Torres-Fuentes LG, Silva-de La Fuente MC, Lareschi M (2020). Molecular detection of Rickettsia in fleas from micromammals in Chile. Parasit Vectors.

[45] Barcelo M, Rubio AV, Simonetti JA. Enhancing habitat quality for small mammals at young pine plantations after clearcutting. Hystrix. 2021;32(1) 10.4404/hystrix-00399-2020.

[46] McCleery R, Monadjem A, Conner LM, Austin JD, Taylor PJ. Methods for ecological research on terrestrial small mammals. Maryland: Johns Hopkins University Press; 2022.

[47] Oliveira-Santos LGR, Tortato MA, Graipel ME (2008). Activity pattern of Atlantic Forest small arboreal mammals as revealed by camera traps. J Trop Ecol.

[48] Meek PD, Zewe F, Falzon G (2012). Temporal activity patterns of the swamp rat (*Rattus lutreolus*) and other rodents in north-eastern New South Wales, Australia. Aust Mammal.

[49] Laughlin MM, Martin JG, Olson ER (2020). Arboreal camera trapping reveals seasonal behaviors of Peromyscus spp in Pinus strobus canopies. Am Midl Nat.

[50] Gracanin A, Mikac KM. Camera traps reveal overlap and seasonal variation in the diel activity of arboreal and semi-arboreal mammals. Mamm Biol. 2022:1–15 10.1007/s42991-021-00218-y.

[51] Li D, Hao J, Yao X, Liu Y, Peng T, Jin Z, Meng F (2020). Observations of the foraging behavior and activity patterns of the Korean wood mouse, *Apodemus peninsulae*, in China, using infra-red cameras. ZooKeys.

[52] Randler C, Kalb J (2020). Predator avoidance behavior of nocturnal and diurnal rodents. Behav Processes.

[53] Rendall AR, Sutherland DR, Cooke R, White J (2014). Camera trapping: a contemporary approach to monitoring invasive rodents in high conservation priority ecosystems. PLoS One.

[54] Gálvez N, Meniconi P, Infante J, Bonacic C (2021). Response of mesocarnivores to anthropogenic landscape intensification: activity patterns and guild temporal interactions. J Mammal.

[55] Scheibler E, Wollnik F, Brodbeck D, Hummel E, Yuan S, Zhang F-S (2013). Species composition and interspecific behavior affects activity pattern of free-living desert hamsters in the Alashan Desert. J Mammal.

[56] Zar JH (1999). Biostatistical analysis.

[57] Batschelet E (1981). Circular statistics in biology.

[58] Kovach W (2011). Oriana-Circular statistics for Windows.

[59] Castro-Arellano I, Lacher TE, Willig MR, Rangel TF (2010). Assessment of assemblage-wide temporal niche segregation using null models. Methods Ecol Evol.

[60] Feinsinger P, Spears EE, Poole RW (1981). A simple measure of niche breadth. Ecology.

[61] Delibes-Mateos M, Díaz-Ruiz F, Caro J, Ferreras P (2014). Activity patterns of the vulnerable guiña (*Leopardus guigna*) and its main prey in the Valdivian rainforest of southern Chile. Mamm Biol.

[62] Whisson DA, Quinn JH, Collins KC (2007). Home range and movements of roof rats (*Rattus rattus*) in an old-growth riparian forest, California. J Mammal.

[63] Murúa R, González L, Meserve P (1986). Population ecology of *Oryzomys longicaudatus philippii* (Rodentia: Cricetidae) in southern Chile. J Anim Ecol.

[64] Meserve PL, Lang BK, Murua R (1991). Characteristics of a terrestrial small mammal assemblage in a temperate rainforest in Chile. Rev Chil Hist Nat.

[65] Meserve PL, Martínez DR, Rau JR, Murúa R, Lang BK, Muñoz-Pedreros A (1999). Comparative demography and diversity of small mammals in precordilleran temperate rainforests of southern Chile. J Mammal.

[66] Shepherd JD, Ditgen RS (2016). Small mammals and microhabitats in Araucaria forests of Neuquén, Argentina. Mastozool Neotrop.

[67] Murúa R, Meserve PL, González LA, Jofré C (1987). The small mammal community of a Chilean temperate rain forest: lack of evidence of competition between dominant species. J Mammal.

[68] Murúa R, González LA (1982). Microhabitat selection in two Chilean cricetid rodents. Oecologia.

[69] Kelt DA, Meserve PL, Lang BK (1994). Quantitative habitat associations of small mammals in a temperate rainforest in southern Chile: empirical patterns and the importance of ecological scale. J Mammal.

[70] Silva SI (2005). Posiciones tróficas de pequeños mamíferos en Chile: una revisión. Rev Chil Hist Nat.

[71] Polop F, Sepúlveda L, Pelliza Sbriller A, Polop J, Provensal MC (2015). Estructura de la dieta de roedores sigmodontinos en arbustales del ecotono bosque-estepa del suroeste de Argentina. Mastozool Neotrop.

[72] Meserve PL, Lang BK, Patterson BD (1988). Trophic relationships of small mammals in a Chilean temperate rainforest. J Mammal.

[73] González L, Murúa R, Jofré C (1989). The effect of seed availability on population density of *Oryzomys* in southern Chile. J Mammal.

[74] Shepherd JD, Ditgen RS (2013). Rodent handling of Araucaria araucana seeds. Austral Ecol.

[75] Meserve PL (1981). Trophic relationships among small mammals in a Chilean semiarid thorn scrub community. J Mammal.

[76] Glanz WE, Meserve PL (1982). An ecological comparison of small mammal communities in California and Chile.

[77] Polop F, Sepúlveda L, PellizaSbriller A, Polop J, Provensal MC (2014). Food habits of Oligoryzomys longicaudatus (Rodentia) in a steppe-forest transitional area of Argentinean Patagonia. Ecol Austral.

[78] Cox MP, Cox CRD, G W. Use of habitat by the black rat (*Rattus rattus*) at North Head, New South Wales: an observational and experimental study. Austral Ecol. 2000;25(4):375–85 10.1046/j.1442-9993.2000.01050.x.

[79] Williams RL, Singleton GR, Dickman CR, Singleton GR, Hinds LA, Krebs CJ, Spratt DM (2003). The role of interspecific competition in determining macrohabitat use by the black rat and brown rat at Bradley's Head, NSW. Rats.

[80] Cole FR, Loope LL, Medeiros AC, Howe CE, Anderson LJ (2000). Food habits of introduced rodents in high-elevation shrubland of Haleakala National Park, Maui, Hawai'i. Pac Sci.

[81] Sweetapple PJ, Nugent G (2007). Ship rat demography and diet following possum control in a mixed podocarp—hardwood forest. N Z J Ecol..

[82] Shiels AB, Flores CA, Khamsing A, Krushelnycky PD, Mosher SM, Drake DR (2013). Dietary niche differentiation among three species of invasive rodents (Rattus rattus, R. exulans, Mus musculus). Biol Invasions.

[83] Stapp P (2002). Stable isotopes reveal evidence of predation by ship rats on seabirds on the Shiant Islands, Scotland. J Appl Ecol.

[84] Guzmán J, Espinoza N, Verdugo N, editors. Predation by *Rattus rattus* on the Hantavirus reservoirs rodent, *Oligoryzomys longicaudatus* in Laguna del Laja National Park, Chile. 6th International Conference of Rodent Biology and Management & 16th Rodens et Spatium; Potsdam, Germany. 2018.

[85] Russell JC, Clout MN (2004). Modelling the distribution and interaction of introduced rodents on New Zealand offshore islands. Global Ecol Biogeogr.

[86] Shiels AB. Ecology and impacts of introduced rodents (*Rattus spp.* and *Mus musculus*) in the Hawaiian islands. Manoa: Dissertation, Department of Botany, University of Hawaii; 2010.

[87] Stokes VL, Banks PB, Pech RP, Spratt DM. Competition in an invaded rodent community reveals black rats as a threat to native bush rats in littoral rainforest of south-eastern Australia. J Appl Ecol. 2009:1239–47 10.1111/j.1365-2664.2009.01735.x.

[88] Harris DB, Macdonald DW (2007). Interference competition between introduced black rats and endemic Galápagos rice rats. Ecology.

[89] Polop F, Levis S, Pini N, Enría D, Polop J, Provensal MC (2018). Factors associated with hantavirus infection in a wild host rodent from Cholila, Chubut Province, Argentina. Mamm Biol.

[90] Mills JN, Ksiazek TG, Peters C, Childs JE (1999). Long-term studies of hantavirus reservoir populations in the southwestern United States: a synthesis. Emerg Infect Dis.

[91] Polop FJ, Provensal MC, Pini N, Levis SC, Priotto JW, Enría D (2010). Temporal and spatial host abundance and prevalence of Andes Hantavirus in Southern Argentina. EcoHealth.

[92] Piudo L, Monteverde MJ, Walker RS, Douglass RJ (2011). Rodent community structure and Andes virus infection in sylvan and peridomestic habitats in northwestern Patagonia, Argentina. Vector Borne Zoonotic Dis.

[93] Torres-Pérez F, Boric-Bargetto D, RE PV. Hantavirus in Chile: New rodents with potential epidemiological importance. Rev Med Chil. 2016;144(6):818. 10.4067/s0034-98872016000600020. 10.4067/S0034-98872016000600020 27598505

[94] Rubio AV, Fredes F, Simonetti JA (2019). Exotic *Pinus radiata* plantations do not increase Andes Hantavirus prevalence in rodents. EcoHealth.

[95] Moreno-Salas L, Espinoza-Carniglia M, Lizama NS, Torres L, la Fuente Silva-de M, Lareschi M (2019). Fleas of black rats (Rattus rattus) as reservoir host of Bartonella spp. in Chile. PeerJ.

[96] Landaeta-Aqueveque C, Salas LM, Henríquez A, Silva-de la Fuente MC, González-Acuña D. Parasites of native and invasive rodents in Chile: ecological and human health needs. Front Vet Sci. 2021;8 10.3389/fvets.2021.643742. 10.3389/fvets.2021.643742 PMC7905021 33644158

[97] Correa JP, Bucarey SA, Cattan PE, Landaeta-Aqueveque C, Ramírez-Estrada J (2017). Renal carriage of Leptospira species in rodents from Mediterranean Chile: The Norway rat (*Rattus norvegicus*) as a relevant host in agricultural lands. Acta Trop.

[98] Kelly DW, Paterson RA, Townsend CR, Poulin R, Tompkins DM (2009). Parasite spillback: a neglected concept in invasion ecology?. Ecology.

[99] Palmeirim AF, Benchimol M, Peres CA, Vieira MV (2019). Moving forward on the sampling efficiency of neotropical small mammals: insights from pitfall and camera trapping over traditional live trapping. Mamm Res.

[100] Dizney L, Dearing MD (2016). Behavioural differences: a link between biodiversity and pathogen transmission. Anim Behav.

[101] Dizney L, Dearing MD (2013). The role of behavioural heterogeneity on infection patterns: implications for pathogen transmission. Anim Behav.

