# Supplementary material for: Activity patterns and interactions of rodents in an assemblage composed by native species and the introduced black rat: implications for pathogen transmission
Source: BMC Zool. 2022 Aug 26;7:48. doi: 10.1186/s40850-022-00152-7 (PMC9412813; doi:10.1186/s40850-022-00152-7)
Supplement: Supplementary file 3 — Additional file 3. [file 40850_2022_152_MOESM3_ESM.docx]

**Supplemental Material**

**Information of the pilot study**

During April 2019, we placed 20 camera traps in the temperate forest located in Huelemolle (39º16 ’S, 71º48’ W), Araucanía Region, southern Chile. During four consecutive days, we tested different positions and configurations of camera traps to improve detection of rodent species. In these tests, the cameras were activated 24 hours a day. There were no videos recorded of rodents during daylight hours. We only obtained recordings during twilight, night, and dawn hours. This is consistent with other previous study conducted by one of the co-authors, where camera traps placed during 2012 and 2013 in several locations in temperate forests of the Araucanía Region did not record rodents during daylight (Gálvez et al. 2021, Journal of Mammalogy 102: 1149-1164. <https://doi.org/10.1093/jmammal/gyab074>).

These results allow as to activate the cameras during crepuscular-nocturnal hours for the study of temporal activity patterns.

**Rodent live-trapping surveys in the study area**

**Table S1**. Number of rodents captured through live trapping using Sherman traps in Huelemolle, Araucanía Region of Southern Chile (Hernández et al 2021 a,b). Rodents were captured in Autumn 2019 (unpublished data) and Winter 2019. Data from the Summer of 2013 correspond to sampling in Huelemolle and nearby areas by Forero (2014).

|  |  | Autumn 2019  (2,268 night-traps) |  | Winter 2019  (1,134 night-traps) |  | Summer 2013  (637 night-traps) |  |  |  |
| --- | --- | --- | --- | --- | --- | --- | --- | --- | --- |
|  | N | Trap success* | N | Trap success* | N | Trap success* |  |  |  |
| *Abrothrix hirta* | 185 | 8.16 | 14 | 1.23 | 15 | 2.36 |  |  |  |
| *Abrothrix olivaceus* | 49 | 2.16 | 8 | 0.71 | 9 | 1.41 |  |  |  |
| *Oligoryzomys longicaudatus* | 53 | 2.34 | 88 | 7.76 | 12 | 1.88 |  |  |  |
| *Rattus rattus* | 5 | 0.22 | 13 | 1.15 | 10 | 1.57 |  |  |  |

*Trap success (no. animal captures/trap night X 100)

Forero, L. 2014. Presence and abundance of *Rattus rattus* and its relationship with composition and structure of the assemblage of native small mammals in the temperate Andean rainforest in southern Chile. Master in Natural Resources, Pontifical Catholic University of Chile.

Hernández, M. C., Jara-Stapfer, D. M., Muñoz, A., Bonacic, C., Barja, I., Rubio, A. V. 2021a. Behavioral responses of wild rodents to owl calls in an Austral temperate forest. Animals, 11: 428.

Hernández, M. C., Rubio, A. V., Barja, I. 2021b. Long-tailed pygmy rice rats modify their behavioural response and faecal corticosterone metabolites in response to culpeo fox but not to lesser grison. Animals, 11: 3036.
